# Carrier multiplication in van der Waals layered transition metal dichalcogenides

**DOI:** 10.1038/s41467-019-13325-9

**Published:** 2019-12-02

**Authors:** Ji-Hee Kim, Matthew R. Bergren, Jin Cheol Park, Subash Adhikari, Michael Lorke, Thomas Frauenheim, Duk-Hyun Choe, Beom Kim, Hyunyong Choi, Tom Gregorkiewicz, Young Hee Lee

**Affiliations:** 1 IBS Center for Integrated Nanostructure Physics (CINAP), Institute for Basic Science, Suwon, 16419 Korea; 2 Department of Energy Science, Sungkyunkwan University, Suwon, 16419 Korea; 3 Institut fur Theoretishe Physik, Universitat Bremen, P. O. Box 330 440, 28334 Bremen, Germany; 4 Bremen Center for Computational Materials Science, Institut fur Theoretische Physik, Universitat Bremen, P. O. Box 330 440, 28334 Bremen, Germany; 5 Inorganic Materials Lab, Samsung Advanced Institute of Technology (SAIT), Suwon, Republic of Korea; 6 School of Electrical and Electronic Engineering, Yonsei University, Seoul, 120-749 Korea; 7 Department of Physics and Astronomy, Seoul National University, Seoul, 08826 Korea; 8 Van der Waals-Zeeman Institute, University of Amsterdam, P.O. Box 94485, 1090 GL Amsterdam, The Netherlands; 9 Present Address: UbiQD, Inc., Los Alamos, NM 87544 USA

**Keywords:** Energy harvesting, Two-dimensional materials, Ultrafast photonics

## Abstract

Carrier multiplication (CM) is a process in which high-energy free carriers relax by generation of additional electron-hole pairs rather than by heat dissipation. CM is promising disruptive improvements in photovoltaic energy conversion and light detection technologies. Current state-of-the-art nanomaterials including quantum dots and carbon nanotubes have demonstrated CM, but are not satisfactory owing to high-energy-loss and inherent difficulties with carrier extraction. Here, we report CM in van der Waals (vdW) MoTe_2_ and WSe_2_ films, and find characteristics, commencing close to the energy conservation limit and reaching up to 99% CM conversion efficiency with the standard model. This is demonstrated by ultrafast optical spectroscopy with independent approaches, photo-induced absorption, photo-induced bleach, and carrier population dynamics. Combined with a high lateral conductivity and an optimal bandgap below 1 eV, these superior CM characteristics identify vdW materials as an attractive candidate material for highly efficient and mechanically flexible solar cells in the future.

## Abstract (web summary)

During carrier multiplication, high-energy free carriers in a given material relax by generation of additional electron-hole pairs. Here, the authors report evidence of carrier multiplication in multilayer MoTe_2_ and WSe_2_ films with up to 99% conversation efficiency.

## Introduction

Atomically thin van der Waals (vdW) layered materials are intensively investigated owing to their fascinating properties. These include exceptional mechanical flexibility and a large range of available band structures and band gaps, from true insulators (hexagonal boron nitride) down to semiconductors (transition metal dichalcogenides, TMDs) and metals (graphene). The TMD-based vdW-layered materials can also have excellent optoelectronic properties, including high in-plane charge carrier mobility. In addition, the very weak vdW bonding between individual layers allows for easy formation of multi-layered structures using the same or different materials, as the lattice matching is not important and a heterostructure with a near-perfect interface may be formed. This new dimension of nanomaterial engineering offers the possibility for a new generation of highly efficient and mechanically flexible optoelectronics. These also include thin-film, mechanically flexible solar cells, owing to band gap tunability by composition and layer thickness, high mobility and a possibility of an ultrahigh internal radiative efficiency of >99%^1^, promoted by good surface passivation and large exciton binding energy. Moreover, absorption of sunlight in semiconducting TMDs monolayers reaches typically 5–10%^2,3^, which is an order of magnitude larger than that in most common photovoltaic materials of Si, CdTe, and GaAs^4^. Accordingly, prototype ultrathin photovoltaic devices, a few atomic layers in thickness, have been realized using MoS_2_ and WSe_2_^5^.

**Fig. 1:**
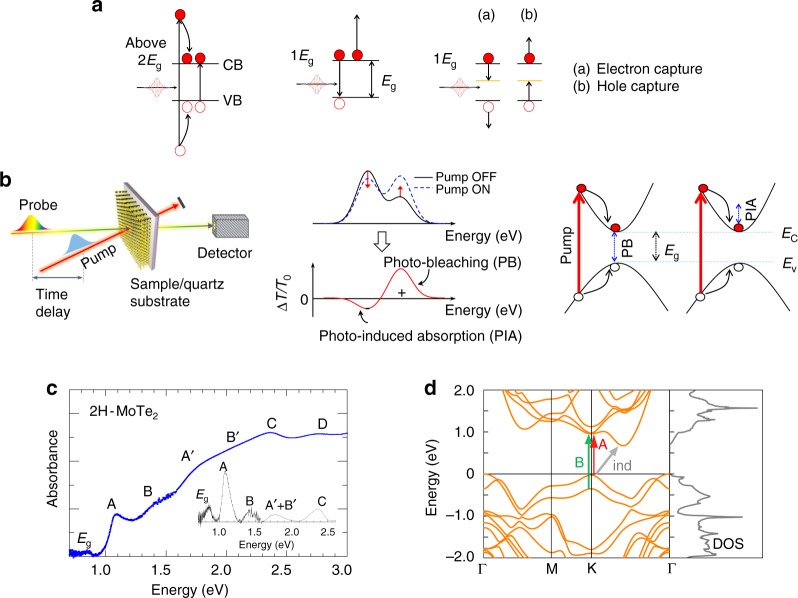
Pump-probe spectroscopy of carrier multiplication (CM). **a** The CM process (left), and two different Auger processes identified in vdW materials. **b** Schematic of the differential transmittance experiment. **c** Steady-state absorption spectrum of the investigated 2H-MoTe_2_ thin film, featuring multiple peaks, including the primary A and B excitons. An indirect bandgap (*E*_g_) is also marked. In the inset, the smooth background absorption has been subtracted, to better reveal the peaks at excitonic transitions. **d** Band structure and density of states for 2H-MoTe_2_ thin-film.

For a single photon absorption in a semiconductor, one photoexcited electron–hole pair is created and yields initial nonthermal distribution of a photoexcited carrier. The photoexcited carrier then interacts with phonon, losing energy to the lattice as heat, which is responsible for intervalley scattering of the carrier. Carrier–carrier scattering mediated by Coulomb interaction redistributes the electron energy to form a quasi-equilibrium state, which follows Fermi–Dirac distribution, responsible for intravalley scattering of the photoexcited carrier. When the excess energy of photoexcited carrier increases above the bandgap energy with strong Coulomb interaction, the photoexcited carrier obtains sufficient energy to scatter with an electron in the valence band, consequently exciting an additional electron across the bandgap to the conduction band, which is known as carrier multiplication (CM, see Fig. 1). CM owing to this inverse Auger process has been suggested to enhance the efficiency of a solar cell above the Shockley−Queisser limit^6^ up to ~46%^7,8^. In bulk semiconductors, the CM process is rather inefficient and has a high threshold energy—typically exceeding 4–5 times the bandgap (*E*_g_)^9,10^. This is due to the low density of final states, limited by the momentum conservation rule, and the rapid carrier cooling by phonon scattering. The situation is different in nanostructures, where quantum confinement relaxes strict momentum conservation and could also affect carrier thermalization^10–13^, which competes with CM^14^.

In general, the conversion efficiency and the threshold energy of CM in a particular material are influenced by (i) Coulomb interactions, which can be promoted by spatial confinement and dielectric screening, (ii) carrier cooling by phonon scattering, (iii) initial/final density of states, and possibly also (especially for thin films), and (iv) surface/defect trapping. The CM conversion efficiency can be promoted by (i) strong Coulomb interaction within spatially confined atomically thin (3–4 Å) layers, (ii) large exciton-binding energies of several hundred meV, much larger than those in quantum wells, and (iii) predominant lower carrier cooling rates by less electron–phonon coupling efficiency due to predominant high exciton binding energies^15–26^. Therefore TMDs, featuring bandgaps in the 0.7–1 eV^8^ range, optimal for CM, could open a possibility by way of increasing the power conversion efficiency of solar cells above the Shockley–Queisser limit.

Here, we report on the observation of the CM phenomenon in thin TMD films of 2H-MoTe_2_ and 2H-WSe_2_. We demonstrate a small CM threshold energy, as low as twice the bandgap, and a high CM conversion efficiency of nearly 93%, when using the usual modeling^11^. These characteristics are superior to those obtained previously for CM in nanostructures and in bulk materials^9,10,12–14,27^. We introduced ultrafast transient absorption spectroscopy with three independent approaches: (i) photo-induced bleaching of the band-to-band absorption at the direct bandgap, (ii) photo-induced intraband absorption of free carriers, and (iii) carrier population dynamics as a function of pump photon energy. These three independent approaches yield consistent results of the CM phenomenon. Because MoTe_2_ possesses a bandgap of 0.85 eV, ideal for CM in solar cells, the current findings identify this material as a strong candidate to maximize the power conversion efficiency in photovoltaics. More generally, these results demonstrate that thin TMD layers have a great potential for advanced light-harvesting technologies and, in particular, for the next generation of highly efficient and mechanically flexible, thin-film solar cells.

## Results

### Transient absorption spectroscopy

In the past, transient absorption spectroscopy, which monitors the optically generated carrier dynamics and utilizes interband and intraband transitions, has been successfully applied to evaluate the CM conversion efficiency in semiconductor quantum dots (QDs)^28,29^. The transient absorption measurement is schematically illustrated in Fig. 1b. A strong pump pulse, with a broadly tunable energy domain, excites carriers from the valence to the conduction band. A weak probe pulse monitors the transmission change with the pump-probe delay time. The photo-induced bleach (PIB), of specific resonant interband transitions, and photo-induced absorption (PIA) owing to intraband excitation of photo-generated free carriers are independently followed. Dynamics of both PIB and PIA signals are monitored by tuning the pump-probe delay time. The differential transmittance, Δ*T*/*T*_0_, in frequency domain is directly measured and is defined as Δ*T*/*T*_0_ = (*T*_on_ − *T*_off_)/*T*_off_, where *T*_on_ and *T*_off_ are the transmission of the probe with and without pump, respectively. The Δ*T/T*_0_ signal represents information on carrier population, either in the specifically probed state (PIB) or within the whole band (PIA).

### Synthesized sample characterization

**Fig. 2:**
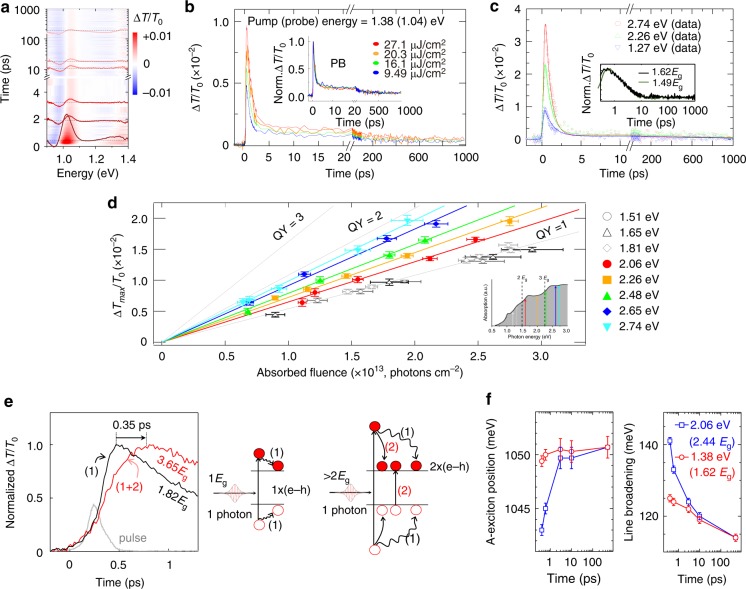
Carrier kinetics in 2H-MoTe_2_ thin film investigated by photoinduced bleaching (PIB). **a** Spectrally and temporally resolved transient absorption map with a pump photon energy of 1.38 eV and a pump fluence of 27.1 μJ cm^−2^. **b** Kinetics of PIB at different photon fluencies with a pump photon energy of 1.38 eV, in the linear regime. The kinetics are invariant when normalized—see the inset—implying absence of nonlinear effects. **c** PB kinetics at different excitation energies (1.27, 2.26, and 2.74 eV), normalized to the equal number of absorbed photons. The solid lines are fitting curves based on bi-exponential and tri-exponential function including the incident Gaussian-shaped pulse. Inset: PIB kinetics at two low excitation photon energies normalized by the absorbed photon density and no noticeable difference between them. **d** The maximum Δ*T*_max_/*T*_0_ intensity as a function of the absorbed photon fluence at different pump energies. The linear slope indicates quantum yield. The steeper the slope, the higher the carrier generation yield. **e** Rise dynamics for two different excitation energies for 2H-MoTe_2_ film. The bleaching signal, taken at direct transition A-exciton, sharply rises at a pump photon energy of 1.82*E*_g_, while the slower rise time is shown at 3.65*E*_g_. The schematic demonstrates the photogenerated carriers with (right) and without (left) CM. The processes (1) and (2) represent hot carrier cooling via phonon emission and carrier multiplication, respectively. **f** Transient Stark shift and biexciton linewidth broadening is shown as a function of delay time.

Our investigations have been performed on a 16.4 nm thin film 2H-MoTe_2_ sample grown by chemical vapor deposition (CVD)^30^. The steady-state absorption spectrum is shown in Fig. 1c (see Supplementary Figs. 1 and 2 for material characterization). We observed two broad peaks, with an energy difference of 330 meV, corresponding to A-exciton and B-exciton transitions (following Wilson and Yoffe’s nomenclature) split by spin–orbit coupling^31^. A direct excitonic bandgap near 1.04 ± 0.03 eV (marked as A in Fig. 1c) and an indirect bandgap of 0.85 ± 0.03 eV (marked as *E*_g_ in Fig. 1c) are in good agreement with the calculated band structure, shown in Fig. 1d, as well as with literature values^32^. The steady-state absorption determines the fraction of absorbed pump photons (under pulsed excitation conditions) (see Supplementary Fig. 3), which can therefore be used for calibration of the absorbed photon density at different excitation energies. The spectrally and temporally resolved Δ*T*/*T*_0_ map is displayed in Fig. 2a, including the TA spectra at different time delays as a function of probe energy; the direct transition for A-exciton can be distinguished as photoinduced bleaching. In addition, the weak B-exciton direct transition is still visible although those peaks are rather broad in energy, and both negative PIA and positive PIB contributions can be observed regardless of pump photon energy (see also Supplementary Fig. 4).

### Photo-induced bleaching at direct bandgap

To investigate CM, we first examine the excitation-photon-energy dependence of the PIB signal^4^. For that purpose, we set the probe energy to the direct bandgap, A-exciton at 1.04 eV. First, we check the fluence-dependent PIB dynamics in the thin film using a pump photon energy of 1.38 eV (900 nm, 1.62*E*_g_) for which CM is not possible. The maximum Δ*T*/*T*_0_, corresponding to the maximum density of carrier population at the probed state, increases linearly with the pump photon density up to 1 × 10^14^ cm^−2^ (equivalently pump fluence of ~22 μJ cm^−2^) when the saturation sets in, as shown in Supplementary Fig. 5. Further, in the linear regime the decay dynamics remain identical. This is illustrated in Fig. 2b by comparison of low-fluence kinetics (pumped at 1.38 eV): the amplitude of the differential transmittance varies with pump fluence (the main panel) but after normalization, all transients collapse into the same curve (inset of Fig. 2b). We conclude that within the linear excitation regime, the amplitude of the Δ*T*/*T*_0_ PIB signal at any delay time is proportional to the number of generated carriers. Therefore, we compare the carrier generation rates at different pump photon energies with a fixed absorbed fluence of ~1.19 × 10^19^ m^−3^ to evaluate CM conversion efficiency^9,29,33^ (Fig. 2c). The maximum intensity nearly doubles at excitation energies of 2*E*_g_ < *E* < 3*E*_g_ and triples at excitation energies of 3*E*_g_ < *E* < 4*E*_g_, compared to the maximum intensity at a pump photon energy <2*E*_g_. This reflects clearly a quantum yield (QY) of impact ionization. This is again strongly supported by the fact that the maximum intensity did not alter at excitation energies <2*E*_g_, as demonstrated in the inset. Therefore, we use the maximum differential transmittance as a measure of QY of impact ionization.

### Determination of QY

We now monitor the maximum Δ*T*_max_/*T*_0_ intensity of PIB kinetics, typically obtained within 0.6 ps after photoexcitation, as a function of fluence at different excitation energies. In each case, we followed the aforementioned outline procedure^3,9,22^ to ensure that the measurements were performed within the linear regime by taking the maximum Δ*T*_max_/*T*_0_ intensity of the PIB signal (Fig. 2d). The maximum intensity increases linearly with the fluence for all excitation energies. As differential transmittance varies with pump photon energy, Δ*T*/*T*_0_ is normalized to the absorbed photon density. As can be seen, upon normalization the experimental points for all excitation energies lower than 2*E*_g_ (1.51, 1.65, and 1.81 eV), fall into a single linear slope. This implies that carrier generation yield is similar for the pump photon energy range, in agreement with similar investigations of CM in other materials^4–6,23^. As CM is not possible for excitation energies below 2*E*_g_, we define this linear slope as corresponding to the carrier generation QY of 1. We now continue the investigations for higher excitation energies and observe a steeper linear slope^10^. Δ*T*_max_/*T*_0_ is related to the QY via absorbed fluence (*F*_abs_); Δ*T*_max_/*T*_0_ = *φσ*_PIB_*F*_abs_, where the proportionality constants are the absorption cross section of the probe (*σ*_PIB_) and the carrier generation QY (*φ*)^9^. The absorption cross section *σ*_PIB_ has been experimentally determined for the low pump photon energy range—*E*_pump_ < 2*E*_g_ and *φ* = 1 by averaging results obtained excitation energies of 1.31, 1.38, 1.46, 1.51, and 1.65 eV, i.e., the QY = 1 level has been arbitrarily set for the below-CM threshold pumping. Using the above relation, the absorption cross-section was found to be *σ*_PIB_ = 5.4 ± 0.05 × 10^−16^ cm^2^. With this value, we then determined *φ* for higher energies by linear fitting of Δ*T*_max_/*T*_0_ vs. *F*_abs_ for each pump photon energy. The slope reaches the QY of *φ* = 2 at the pump photon energy of 2.74 eV.

### Carrier population dynamics

For further analysis of free carrier population with the build-up dynamics^28,34^, we consider the rise time of PIB signal with a higher temporal resolution of 50 fs at 1.55 (<2*E*_g_) and 3.1 eV (>2*E*_g_) pump energies (see SI Method). The bleach, measured at the direct transition of A-exciton (see Fig. 2e), rises quickly (~0.4 ps) and is saturated for <2*E*_g_ owing to hot carrier relaxation (see process (1) in the scheme of Fig. 2d). For the pump photon energy of >2*E*_g_, we observe a relatively slow (~0.75 ps) and featureless rise time, which is attributed to hot carrier cooling and CM—see the respective processes (1) and (2). This slower rise time at the higher pump photon energy could be explained by the larger excess energy of the photoexcited carrier, resulting in longer intravalley scattering towards the conduction band edge which is probed by PIB signal. We can neglect such a probability because a similar aspect, i.e., slow rise time at a higher excitation photon energy, is also observed in PIA signal, which will be discussed later. Therefore, this comparison of the PIB rise time for below-CM and above-CM threshold pumping provides very clear and independent fingerprint of CM^28,34^. Nevertheless, other possible scenarios for the slow rise time including the thermalization process of hot carriers towards the band edge, the state filling time by the carrier population, and the scattering in another valley cannot be excluded.

### The fingerprint of CM

When CM occurs, one photoexcited exciton and additionally excited exciton give rise to the formation of biexciton^28,29,35–39^. As the photoexcited carriers increase, a strong local field is developed, consequently predominating a transient Stark shift and a broadened linewidth of absorbance^28,35–38^. Therefore, the presence of biexciton state will be a monitor of CM at above threshold pumping^29,35,37^. We compared the pump-excited absorbance spectra for excitation at >2*E*_g_ to absorbance spectrum without pumping (Supplementary Fig. 4), and clearly observed two evidences of CM (Fig. 2f). The spectrum at early time delay (0.4 ps) is red-shifted by ~9 meV with respect to one at a late time delay (>500 ps), originating from the Stark shift. We simultaneously observed that the spectral shape and amplitude of the differential signal at >2*E*_g_ were overlapped with those of <2*E*_g_, confirming the CM evidence (Supplementary Fig. 4). Moreover, the linewidth at early time delay is also broadened by ~145 meV, compared to that at the late time delay. These spectral shift and line-broadening in the time evolution provide evidence of our CM results, corresponding with the population dynamics shown in Fig. 2e.

### Photo-induced intraband absorption of free carriers

**Fig. 3:**
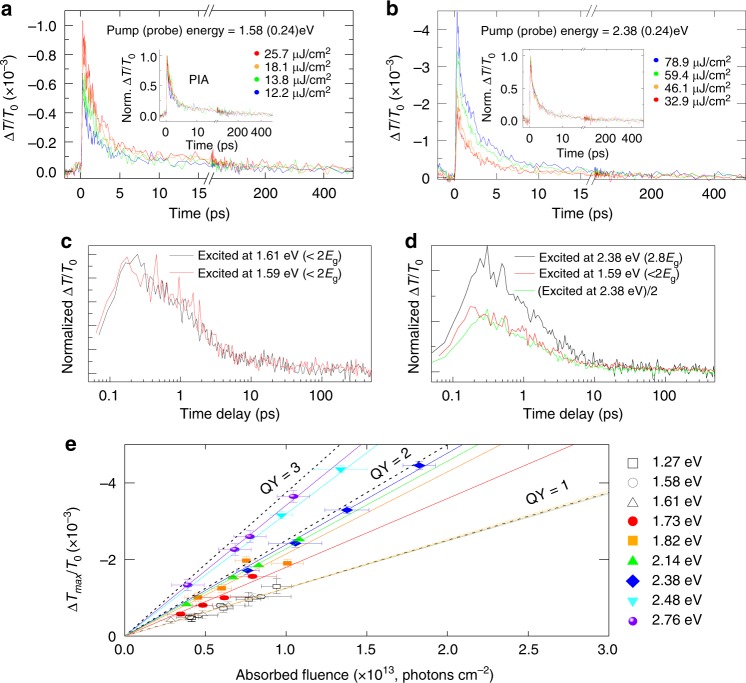
Carrier kinetics in 2H-MoTe_2_ thin film investigated by photo-induced absorption (PIA). Kinetics of PIA at different photon fluencies with a pump photon energy of **a** 1.58 eV and **b** 2.38 eV. The kinetics are invariant when normalized—see the inset—implying absence of nonlinear effects. **c** Photo-induced absorption kinetics at two low excitation photon energies normalized by the absorbed photon density and no noticeable difference in the trace is observed. **d** PIA kinetics excited at 2.38 eV (2.8*E*_g_, black) and 1.58 eV (1.86*E*_g_, red), normalized to the equal number of absorbed photon density. PIA kinetics excited at 2.38 eV is scaled by a factor of 0.5. **e** The maximum Δ*T*_max_/*T*_0_ intensity extracted from the kinetics of photoinduced absorption as a function of absorbed fluence at different pump photon energies. The linear slope indicates quantum yield. The steeper the slope of the line, the higher the carrier generation yield.

As mentioned before, the concentration of photogenerated free carriers can also be inferred from their absorption. Therefore, CM can be independently investigated from pump photon energy dependence of PIA. In this case, the PIA signal is obtained by probing intraband transitions of free carriers^10,11^. Therefore, this approach is slightly different from the PIB which probes a specific state (A-exciton in the previously considered via PIB) because the measured PIA represents the response of all the free carriers present in the sample. The advantage of probing PIA in the infrared energy over probing PIB at the band edge energy is that the transient kinetics of PIA preserves the linearity to high carrier number, which are independent of the probe wavelength^28,34,40,41^. A probe energy of 0.24 eV (5200 nm) has been chosen because of the superior signal to noise ratio of the differential PIA measurement, but that particular choice is rather indifferent to the determination of CM conversion efficiency (See Supplementary Fig. 6). As with the PIB investigation, we first check the linear response of the system; Fig. 3a, b illustrate low-fluence PIA kinetics obtained for the different pump fluence, with the pump photon energy set at 1.58 and 2.38 eV, i.e., below 2*E*_g_ and above 2*E*_g_, respectively. Again, when normalized to the same absorbed photon density, they all coincide, as shown in the inset, implying that the PIA amplitude is proportional to the number of absorbed photons, with no nonlinear effects, which can therefore be used to monitor chances of free carrier concentration. For completeness, we also check that photo-charging effects, which frequently trouble PIA studies, are not present in this case (Supplementary Fig. 7). Subsequently, we measure the PIA kinetics for different excitation energies (Fig. 3c, d) and compare the dependence of their amplitude on the fixed absorbed excitation photon fluence (Fig. 3e). By this way we can compare carrier generation yield at different pump photon energy, as in the PIB analysis. While we chose the maximum Δ*T*/*T*_0_ intensity of the early delay time for QY determination of PIA, a similar QY was obtained at a later delay time (see Supplementary Fig. 8). For this comparison similar to PIB, we choose the maximum Δ*T*/*T*_0_ intensity, directly following the pump pulse because it is most likely to represent the total concentration of free carriers. For completeness, we point out that in this case, even if the PIA signal is actually lowered by an ultrafast carrier recombination, the carrier generation yield determined in this way would still lead to feasible CM process.

### CM conversion efficiency

**Fig. 4:**
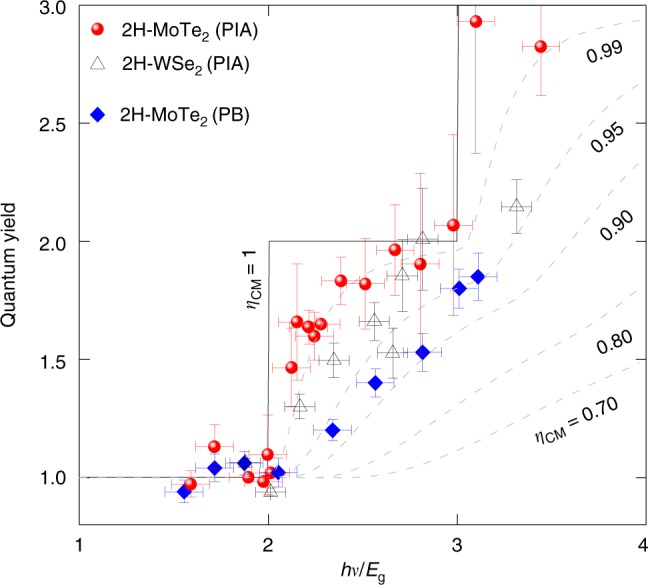
Quantum yield of carrier generation for 2H-MoTe_2_ and 2H-WSe_2_ thin film. **a** The QY data for 2H-MoTe_2_ film (blue diamonds by PB method and red dots by PIA method) are plotted as a function of pump photon energy normalized by the bandgap of material. The black solid line and the dashed lines in gray color represent simulations of CM efficiency (*η*_CM_).

Figure 4 shows the determined carrier generation QY (blue diamonds and red dots for PIB and PIA approach, respectively), as a function of the pump photon energy normalized to the bandgap energy of 2H-MoTe_2_. A step-like onset of CM at threshold energy just above 2*E*_g_ is clearly manifested, with an abrupt increase of QY for pump photon energies exceeding ~2*E*_g_. To characterize the observed CM process, we use the commonly applied model proposed by Beard et al.^14^, which can be utilized regardless of the electronic band structure. We fit the experimental data to the formula *hν*/*E*_g_ = 1 + *φ*/*η*_CM_, where *hν* is the incident pump photon energy and *η*_CM_ the CM conversion efficiency. The fitting yields efficiency of *η*_CM_ ≈ 95% and ≈99% for the data sets obtained from PIB and PIA approaches, respectively. Alternatively the CM conversion efficiency can be evaluated by comparison of the direct integral of the fitted curves with respect to the step-like characteristics of the ideal CM (Fig. 4); this procedure yields the efficiencies of ~45% and ~70% for PIB and PIA data sets, respectively. We note that the CM characteristics obtained from the PIA and PIB measurements are similar, with the identical offset energy and the absolute efficiency being somewhat higher for PIA. We can rationalize this observation by pointing out that PIA monitors all photo-excited free carriers, whereas PIB only investigated for a particular energy and thus provides information on a population of a particular state—the A-exciton in this case. Therefore, the PIA approach seems to be more suitable than PIB, especially for indirect bandgap materials, but that requires further study.

In an effort to generalize our study, we investigated CM in another 2D vdW material—2H-WSe_2_ film (hollow triangles in Fig. 4) which has an indirect bandgap of 0.9 eV (Supplementary Fig. 9). The measurements have been performed in the PIA mode, in the same way as described before for 2H-MoTe_2_. Highly efficient CM has also been found in this material, with low threshold energy of ~2*E*_g_ and the efficiency of ~97% and 52%, as evaluated using the description by Beard et al.^14^ and by direct integration, respectively. Therefore, the high CM conversion efficiency with a low-energy threshold of ~2*E*_g_ and the step-like characteristics seem likely to be more general for 2D vdW dichalcogenides.

### Decay kinetics of photoexcited states

To independently validate the claim of CM in the 2H-MoTe_2_ vdW-layered material, we examine decay dynamics of the photogenerated carriers. In the past, abrupt change of carrier dynamics when pumped below and above the CM threshold served to identify this phenomenon in QDs of various semiconductors^9,11,12,34,42,43^. Typically, such a change is facilitated by an efficient Auger recombination switching on as soon as multiple excitons appear simultaneously in the same quantum dot. This effect is not expected in vdW layers where multiple photo-generated carriers can effectively separate within the 2D plane, and in that way escape rapid recombination. Nevertheless, understanding the relevant ultrafast kinetics in the material is important to evaluate the signature of CM. Accordingly, we study first the fluence-dependent PIB dynamics using a pump photon energy of 1.38 eV (900 nm, 1.62*E*_g_) in which CM does not occur and in the linear regime, as discussed before. The kinetics can be fitted by double exponential decay, with two components: a fast and a slow one, with time constants of 2.9 ± 0.6 ps (*τ*_f_) and 3 ± 1 s (*τ*_s_), respectively, (see Supplementary Fig. 10a). Based on the literature^27^, we attribute the fast component to the decay of the A-exciton state population to either trap states or mid-gap states. The slow component is of the similar order to the radiative recombination time. When we increase the fluence, entering the saturation range of the PIB signal, an additional fast decay component of ~0.36 ps (*τ*_A_) appears; in a recent report^44^ this fast recombination channel has been assigned to defect-mediated fast Auger recombination process arising in vdW materials at sufficiently high free carrier density. We conclude therefore that carrier decay dynamics in the investigated sample change with concentration, and therefore can be used to probe it.

We next measured the fluence-dependent kinetics excited at 2.06 eV (2.42*E*_g_) (Supplementary Fig. 10b). Except for the very lowest fluence, all the dynamics have three dominant components of 0.31 ± 0.08 ps (*τ*_1_), 3.04 ± 0.6 ps (*τ*_2_), and 2.94 ± 0.7 ns (*τ*_3_). In Supplementary Fig. 10c we directly compare two PIB dynamics: one has been obtained for below CM threshold pumping at 1.38 eV and a relatively high fluence, and the other one for the above CM threshold pump photon energy of 2.06 eV and a lower fluence. As can be seen, the normalized transients are identical within the experimental resolution. Following the earlier reasoning, we conclude that in both cases a similar concentration of free carriers has been obtained; because the absorbed photon flux by the high-energy pumping has been considerably smaller, ~8.75 × 10^18^ vs. ~2.81 × 10^19^ cm^−3^, it confirms a higher carrier generation yield, consistent with the claim of CM. Therefore, the enhancement of the number of carriers generated by a high-energy photon is now supported not only by the amplitude but also by the (concentration-dependent) carrier decay dynamics.

## Discussion

**Fig. 5:**
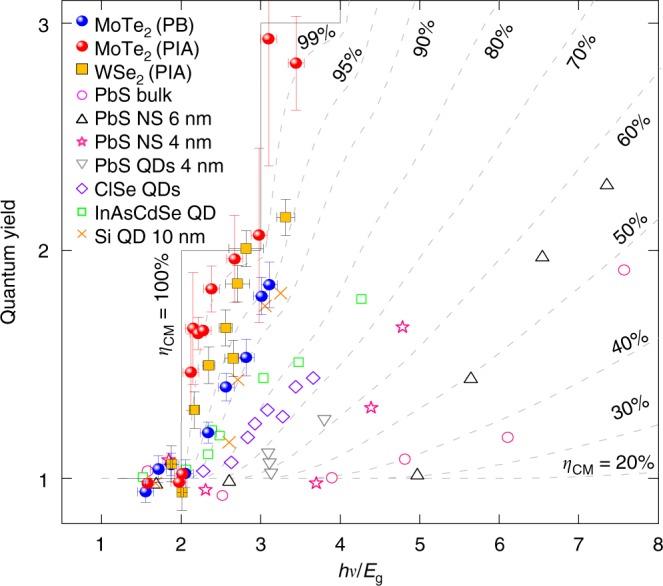
Quantum yield for various nanostructures and bulk materials and the extracted carrier multiplication. Comparison of the CM efficiency of thin-film 2H-MoTe_2_ and 2H-WSe_2_ with various other semiconductors including bulk, QDs, and nanoplatelets. Quantum yields from previous reports were taken from refs. ^9,11–13,42,45,46^.

In Fig. 5, we compare the CM characteristics reported for various semiconductors in different geometries, using the phenomenological description of Beard et al.^14^. In general, the geometry of a material has a dramatic effect on the CM conversion efficiency^9,11–13,42,45,46^. For example, CM in PbS nanoplatelets and QDs is more efficient than in their bulk counter-part^9,13^. Material composition also has a large effect on both threshold energy and CM conversion efficiency. A step-like feature with threshold energy of 2.5*E*_g_ and CM conversion efficiency of 90% has been reported for Si QDs^11^. The most efficient CM materials measured to date have been QDs dots with a threshold energy of over 2.5*E*_g_ or 3*E*_g_ but with the CM efficiency below 90%. Recent reports for PbSe QD films^47^ and improved CM conversion efficiency in nanorods^13,27^, and InAs QDs^42^ show a threshold in the vicinity of 2*E*_g_ but a poor CM conversion efficiency of 35–80%. By contrast, the vdW-layered materials investigated here clearly manifest threshold energies near 2*E*_g_, with no excess energy and the record CM conversion efficiency up to ~99% (using the PIA approach).

The microscopic origin of the high CM conversion efficiency in TMD-based vdW-layered materials is not clear at this point, and will require further investigations and better theoretical understanding of their band structure. As mentioned before, the CM conversion efficiency is governed in principle by the competition between carrier–carrier and carrier–phonon scattering. The electron–electron scattering time constant in 2D materials is considerably shorter than for 1D nanowires and 0D QDs—see Supplementary Table 1. The ratio of time constants of electron–electron and electron–phonon scattering (*τ*_e–e scatt_/*τ*_e–ph scatt_) is about 30. Therefore, we can expect that the carrier–carrier will dominate over carrier–phonon scattering in 2D materials. Consequently, for the high-energy pumping carrier cooling in 2D materials might be governed by CM, dominating phonon scattering. Further, we speculate that the strong confinement effect by the electrostatic potential barrier built in extremely narrow region of 3–4 Å between layers^48^ (Supplementary Fig. 11), as well as specific peculiarities of the density of states (DOS pockets) will play a role, acting as efficiency boosters for the CM process.

In summary, we demonstrate a highly efficient CM in 2H-MoTe_2_ and 2H-WSe_2_ with nearly ideal characteristics: The onset energy close to the energy conservation of twice the bandgap energy and up to ~99% CM conversion efficiency. This is ascribed to the specific features of 2D vdW-layered materials, strong Coulomb interaction, and in particular, the efficient carrier–carrier scattering and peculiarities of the band structure. High conductivity, large absorbance, and optimal bandgap could be a promising 2D vdW 2H-MoTe_2_ material for next-generation flexible and highly efficient solar cells.

## Methods

### Transient absorption spectroscopy

To measure the dynamics of photoexcited carriers, transient absorption spectroscopy was performed. A beam from 1 kHz Ti:sapphire regenerative amplifier (Libra, Coherent) operating at 790 nm was divided by a beam splitter, at the 1:9 ratio. The stronger beam drives two optical parametric amplifiers (TOPAS prime, Light Conversion), one of which generates laser light in the ultraviolet to mid-infrared range. The OPA was used as a tunable pump pulse with visible to near-infrared energies (1.27–3.1 eV) that excited electron–hole pairs in the sample. The weaker beam generates white-light continuum and was used as the probe pulse, and was detected by a CMOS sensor for the visible range, and an InGaAs sensor for the near-infrared range (HELIOS, Ultrafast systems). Another OPA pumped by the amplifier was tuned in the range of 0.23–0.25 eV to probe the photoinduced absorption, and was detected by HgCdTe detector (HELIOS IR, Ultrafast systems). Both pump and probe beams were linearly polarized, and parallel to each other. By inserting a KTA (potassium titanyle arsenate, KTiOAsO_4_) crystal at a sample position, we measured the cross-correlation signal at two different wavelengths (visible for the pump and infrared for the probe), and the measured pulse duration was ~205 ± 20 fs (Supplementary Fig. 12). To measure the rise time component in the transient kinetics, another laser system was used to generate 1.55 and 3.1 eV pump energies with a higher temporal resolution of ~50 fs. The absorbed photon density was determined as the pump power measurement passing through pinholes of 50, 75, and 100 μm at the sample position. Depending on the laser wavelength, the focused pump spot size varied within the range of 200–230 μm, much larger than the 50 μm pinhole diameter. Background absorption of the substrate was subtracted. The fraction of absorbed pump photons, which were obtained from ultrafast pulse at different excitation energies, followed the steady-state absorbance curve obtained for CW source (Supplementary Fig. 3). The error bar in the maximum of the transient absorption signal was determined from the noise level measured at negative time delays in the kinetics for each data set. The error bar in the QY plot was determined from the standard deviation of the energy measurement of the pump fluence.

### 2H-MoTe_2_ thin films

To grow semiconducting 2H molybdenum ditelluride (2H-MoTe_2_) thin films, 7-nm-thick Mo thin film was deposited on a 300-nm-thick SiO_2_/Si substrate via a sputtering system. The prepared Mo thin film was mounted in a two-zone CVD system. The Mo thin film located in the second furnace zone was tellurized by vaporizing 2 g of tellurium (Te) powder (Sigma-Aldrich) at the first furnace zone. To control the tellurization rate, the temperatures of the Te zone (*T*_1_) and Mo film zone (*T*_2_) were controlled independently. During the growth process, argon and hydrogen gases were flown with rates of 500 and 100 sccm, respectively. *T*_1_ was first heated up to 620 °C over 15 min and then *T*_2_ was ramped to 535 °C in 5 min. When the *T*_2_ temperature reached 535 °C, growth was carried out for 5 h. After growth, *T*_1_ was cooled rapidly by opening the chamber; *T*_2_ was then cooled to room temperature at a rate of 50 °C/min^30^.

### Transfer method for 2H-MoTe_2_ film

To transfer the MoTe_2_ film from as-grown substrate to a quartz substrate, the poly (methyl methacrylate) (PMMA) was coated on the MoTe_2_ film surface. The PMMA-coated MoTe_2_ film was dried for more than 30 min in air. After that, the PMMA-coated MoTe_2_ film was floated on a buffered oxide etchant (1178-03, J.T. Baker) to etch the SiO_2_ layer under the MoTe_2_ film. Then, the PMMA/MoTe_2_ film was rinsed with distilled water several times. The product film was transferred onto a target substrate. After drying the film, the PMMA layer was removed with acetone and isopropyl alcohol.

### Sample characterization

The linear absorption spectrum of 2H-MoTe_2_ film was measured by using a UV/VIS absorption spectrometer (V-670, JASCO). The absorbance shown in Fig. 1b was obtained after subtraction of transmittance and reflectance which were measured in an integrating sphere automatically. Raman spectroscopy (Ranishaw) was performed with an excitation energy of 2.33 eV (532 nm). XRD (SmartLab, Rigaku), AFM (SPA400, SEIKO), SEM (FESEM, JSM7000F, JEOL), and TEM (JEM ARM 200F, JEOL Ltd.) were used to characterize the films. We describe the details of each result in Supplementary Figs. 2 and 3.

## Supplementary information

Supplementary Information

## Data Availability

The data that support the findings of this study are available from the corresponding authors upon reasonable request.

## References

[1] Amani M (2015). Near-unity photoluminescence quantum yield in MoS_2_. Science.

[2] Bernardi M, Palummo M, Grossman JC (2013). Extraordinary sunlight absorption and on nanometer thick photovoltaics using two-dimensional monolayer materials. Nano Lett..

[3] Villegas CE, Rocha A (2015). Elucidating the optical properties of novel heterolayered materials based on MoTe2–InN for photovoltaic applications. J. Phys. Chem. C.

[4] Polman A, Knight M, Garnett EC, Ehrler B, Sinke WC (2016). Photovoltaic materials: present efficiencies and future challenges. Science.

[5] Furchi MM, Pospischil A, Libisch F, Burgdörfer J, Mueller T (2014). Photovoltaic effect in an electrically tunable van der Waals heterojunction. Nano Lett..

[6] Shockley W, Queisser HJ (1961). Detailed balance limit of efficiency of p–n junction solar cells. J. Appl. Phys..

[7] Beard MC, Luther JM, Semonin OE, Nozik AJ (2012). Third generation photovoltaics based on multiple exciton generation in quantum confined semiconductors. Accounts Chem. Res..

[8] Hanna MC, Beard MC, Nozik AJ (2012). Effect of solar concentration on the thermodynamic power conversion efficiency of quantum-dot solar cells exhibiting multiple exciton generation. J. Phys. Chem. Lett..

[9] Aerts M (2014). Highly efficient carrier multiplication in PbS nanosheets. Nat. Commun..

[10] Trinh MT (2012). Direct generation of multiple excitons in adjacent silicon nanocrystals revealed by induced absorption. Nat. Photon..

[11] Beard MC (2007). Multiple exciton generation in colloidal silicon nanocrystals. Nano Lett..

[12] Cirloganu CM (2014). Enhanced carrier multiplication in engineered quasi-type-II quantum dots. Nat. Commun..

[13] Padilha LA (2013). Aspect ratio dependence of auger recombination and carrier multiplication in PbSe nanorods. Nano Lett..

[14] Beard MC (2010). Comparing multiple exciton generation in quantum dots to impact ionization in bulk semiconductors: implications for enhancement of solar energy conversion. Nano Lett..

[15] del Corro E (2016). Atypical exciton–phonon interactions in WS_2_ and WSe_2_ monolayers revealed by resonance Raman spectroscopy. Nano Lett..

[16] Koirala S, Mouri S, Miyauchi Y, Matsuda K (2016). Homogeneous linewidth broadening and exciton dephasing mechanism in MoTe_2_. Phys. Rev. B.

[17] Moody G (2015). Intrinsic homogeneous linewidth and broadening mechanisms of excitons in monolayer transition metal dichalcogenides. Nat. Commun..

[18] Komsa H-P, Krasheninnikov AV (2012). Effects of confinement and environment on the electronic structure and exciton binding energy of MoS_2_ from first principles. Phys. Rev. B.

[19] Chernikov A (2014). Exciton binding energy and nonhydrogenic Rydberg series in monolayer WS_2_. Phys. Rev. Lett..

[20] He K (2014). Tightly bound excitons in monolayer WSe_2_. Phys. Rev. Lett..

[21] Yang J (2015). Robust excitons and trions in monolayer MoTe_2_. ACS Nano.

[22] Tielrooij KJ (2013). Photoexcitation cascade and multiple hot-carrier generation in graphene. Nat. Phys..

[23] Thilagam A, Singh J (1993). Generation rate of 2D excitons in quantum wells. J. Lumin..

[24] Thilagam A (2016). Exciton formation assisted by longitudinal optical phonons in monolayer transition metal dichalcogenides. J. Appl. Phys..

[25] Ugeda MM (2014). Giant bandgap renormalization and excitonic effects in a monolayer transition metal dichalcogenide semiconductor. Nat. Mater..

[26] Szczytko J (2004). Determination of the exciton formation in quantum wells from time-resolved interband luminescence. Phys. Rev. Lett..

[27] Cunningham PD (2011). Enhanced multiple exciton generation in quasi-one-dimensional semiconductors. Nano Lett..

[28] Ellingson RJ (2005). Highly efficient multiple exciton generation in colloidal PbSe and PbS quantum dots. Nano Lett..

[29] Schaller RD, Agranovich VM, Klimov VI (2005). High-efficiency carrier multiplication through direct photogeneration of multi-excitons via virtual single-exciton states. Nat. Phys..

[30] Park JC (2015). Phase-engineered synthesis of centimeter-scale 1T’- and 2H-molybdenum ditelluride thin films. ACS Nano.

[31] Ruppert C, Aslan OB, Heinz TF (2014). Optical properties and band gap of single- and few-layer MoTe_2_ crystals. Nano Lett..

[32] Nie Z (2014). Ultrafast Carreir thermalization and cooling dynamics in few-layer MoS_2_. ACS Nano.

[33] Trinh MT (2008). In spite of recent doubts carrier multiplication does occur in PbSe nanocrystals. Nano Lett..

[34] de Weerd C (2018). Efficient carrier multiplication in CsPbI_3_ perovskite nanocrystals. Nat. Commun..

[35] Choi Y, Sim S, Lim SC, Lee YH, Choi H (2013). Ultrafast biexciton spectroscopy in semiconductor quantum dots: evidence for early emergence of multiple-exciton generation. Sci. Rep..

[36] Hu Y (1990). Biexcitons in semiconductor quantum dots. Phys. Rev. Lett..

[37] Gesuele F (2012). Ultrafast supercontinuum spectroscopy of carrier multiplication and biexcitonic effects in excited states of PbS quantum dots. Nano Lett..

[38] Klimov V, Hunsche S, Kurz H (1994). Biexciton effects in femtosecond nonlinear transmission of semiconductor quantum dots. Phys. Rev. B.

[39] Rupasov VI, Klimov VI (2007). Carrier multiplication in semiconductor nanocrystals via intraband optical transitions involving virtual biexciton states. Phys. Rev. B.

[40] Ellingson RJ (2002). Excitation energy dependent efficiency of charge carrier relaxation and photoluminescence in colloidal InP quantum dots. J. Phys. Chem. B.

[41] Cha S (2016). 1s-intraexcitonic dynamics in monolayer MoS2 probed by ultrafast mid-infrared spectroscopy. Nat. Commun..

[42] Schaller RD, Pietryga JM, Klimov VI (2007). Carrier multiplication in InAs nanocrystal quantum dots with an onset defined by the energy conservation limit. Nano Lett..

[43] Binks DJ (2011). Multiple exciton generation in nanocrystal quantum dots—controversy, current status and future prospects. Phys. Chem. Chem. Phys..

[44] Wang H (2015). Fast exciton annihilation by capture of electrons or holes by defects via Auger scattering in monolayer metal dichalcogenides. Phys. Rev. B.

[45] Stolle CJ, Schaller RD, Korgel BA (2014). Efficient carrier multiplication in colloidal CuInSe_2_ nanocrystals. J. Phys. Chem. Lett..

[46] Pijpers JJH (2009). Assessment of carrier-multiplication efficiency in bulk PbSe and PbS. Nat. Phys..

[47] Sandeep CSS (2013). High charge-carrier mobility enables exploitation of carrier multiplication in quantum-dot films. Nat. Commun..

[48] Yu WJ (2016). Unusually efficient photocurrent extraction in monolayer van der Waals heterostructure by tunnelling through discretized barriers. Nat. Commun..

