# Supplementary material for: Carrier multiplication in van der Waals layered transition metal dichalcogenides
Source: Nat Commun. 2019 Dec 2;10:5488. doi: 10.1038/s41467-019-13325-9 (PMC6889496; doi:10.1038/s41467-019-13325-9)
Supplement: Supplementary file 1 — Supplementary Information [file 41467_2019_13325_MOESM1_ESM.pdf]

Supplementary Information for

Carrier Multiplication in van der Waals Layered Transition Metal Dichalcogenides

Kim et al.

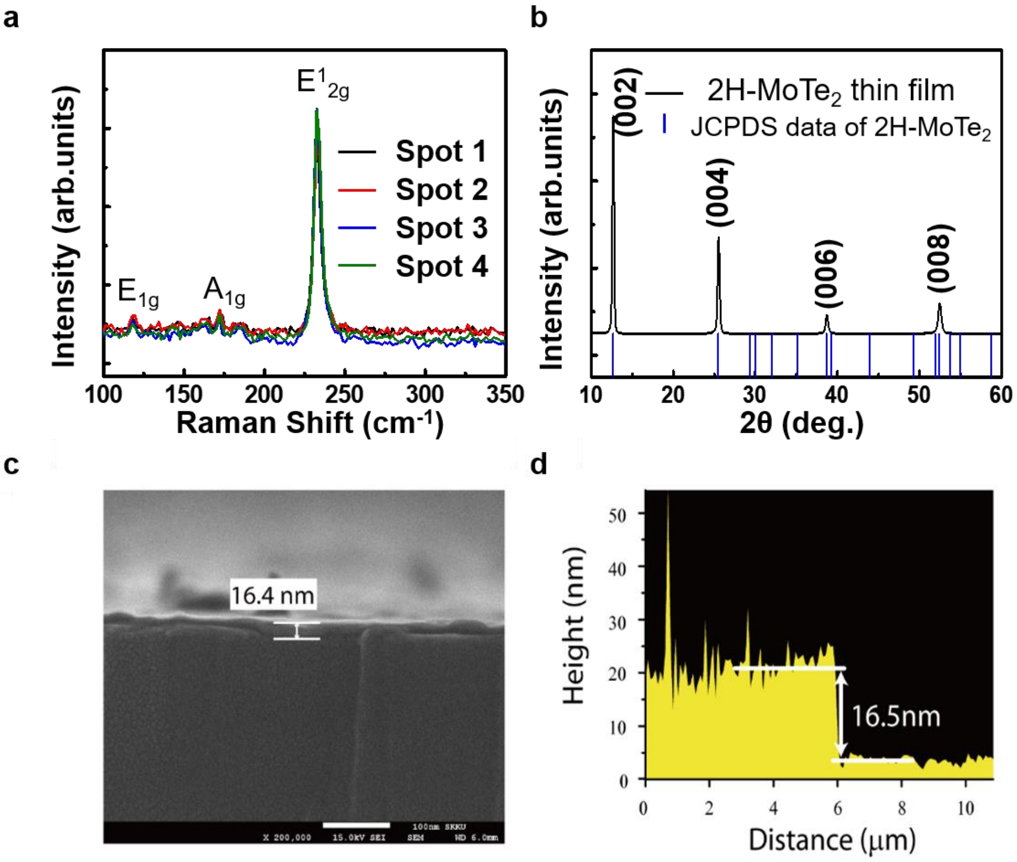

Supplementary Figure 1. **Material properties of 2H-MoTe$_2$ thin film[1]. a**, Raman spectra from several spots of 2H-MoTe$_2$ thin film obtained with an excitation energy of 532 nm, confirming homogeniety of the sample. **b**, XRD pattern of 2H-MoTe$_2$ thin film and Joint Committee on Powder Diffraction Standards (JCPDS) database of 2H-MoTe$_2$. The spectra match with (002), (004), (006), and (008) planes of 2H-MoTe$_2$. **c**, Cross-sectional SEM image of 2H-MoTe$_2$ thin film on SiO$_2$/Si substrate. The thickness of the 2H-MoTe$_2$ thin film is ~16.4 nm. It is well-matched with the height profile obtained with the atomic force microscope in **d**.

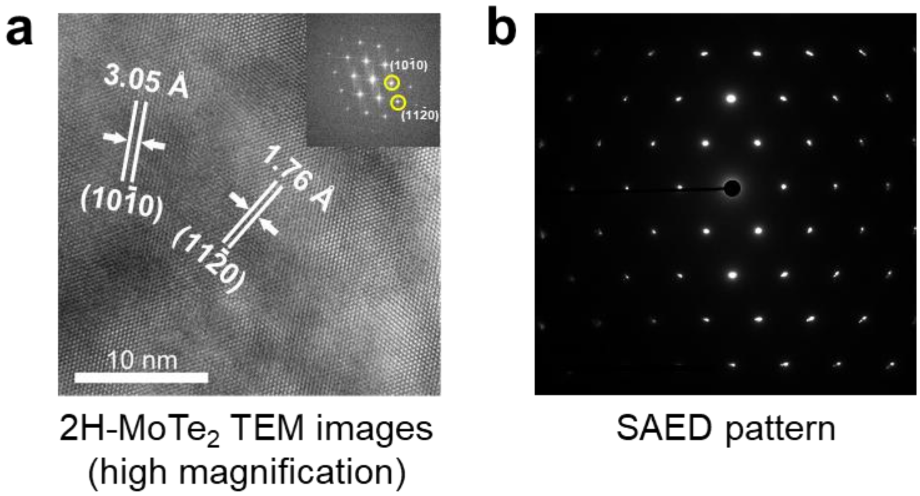

Supplementary Figure 2. **TEM analysis of 2H-MoTe$_2$ thin film.** HRTEM image of **a**, 2H-MoTe$_2$. The inset of image shows the corresponding FFT pattern, confirming the 2H-MoTe$_2$ phase. **b**, Selective area electron diffraction (SAED) pattern of 2H-MoTe$_2$ in **a**. The extracted lattice constants match with the known values of 2H-MoTe$_2$.

To confirm the crystal structure and crystallinity of the 2H-MoTe$_2$ thin film, high-resolution transmission electron microscopy (HR-TEM) was used. Figures S3a and S3b show the HR-TEM image and the corresponding selective-area electron diffraction (SAED) pattern of 2H-MoTe$_2$ thin film, respectively. The inset of Figure S3a displays the fast Fourier transform (FFT) pattern. While the FFT pattern of 1T'-MoTe$_2$ is rectangular, that of the 2H-MoTe$_2$ is hexagonal. Furthermore, the d-spacings of the $(10\bar{1}0)$ and $(11\bar{2}0)$ planes for 2H-MoTe$_2$ are resolved to be 3.05 and 1.76 Å, respectively. These are well-matched with literature values. SAED was adopted to estimate the size of the crystallites of the 2H-MoTe$_2$ thin film. An aperture diameter of 10 μm was used for this study, indicating that the size of crystallites of 2H-MoTe$_2$ thin film was at least 10 μm.

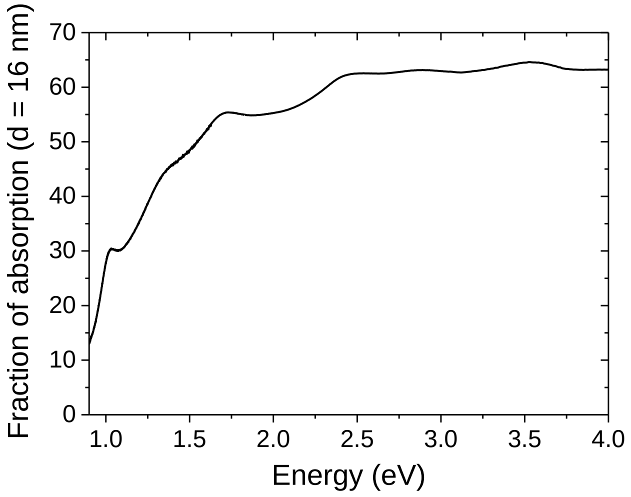

Supplementary Figure 3. Fraction of absorbed light. The fraction of absorbed pump photons, which were obtained from ultrafast pulse at different excitation energies.

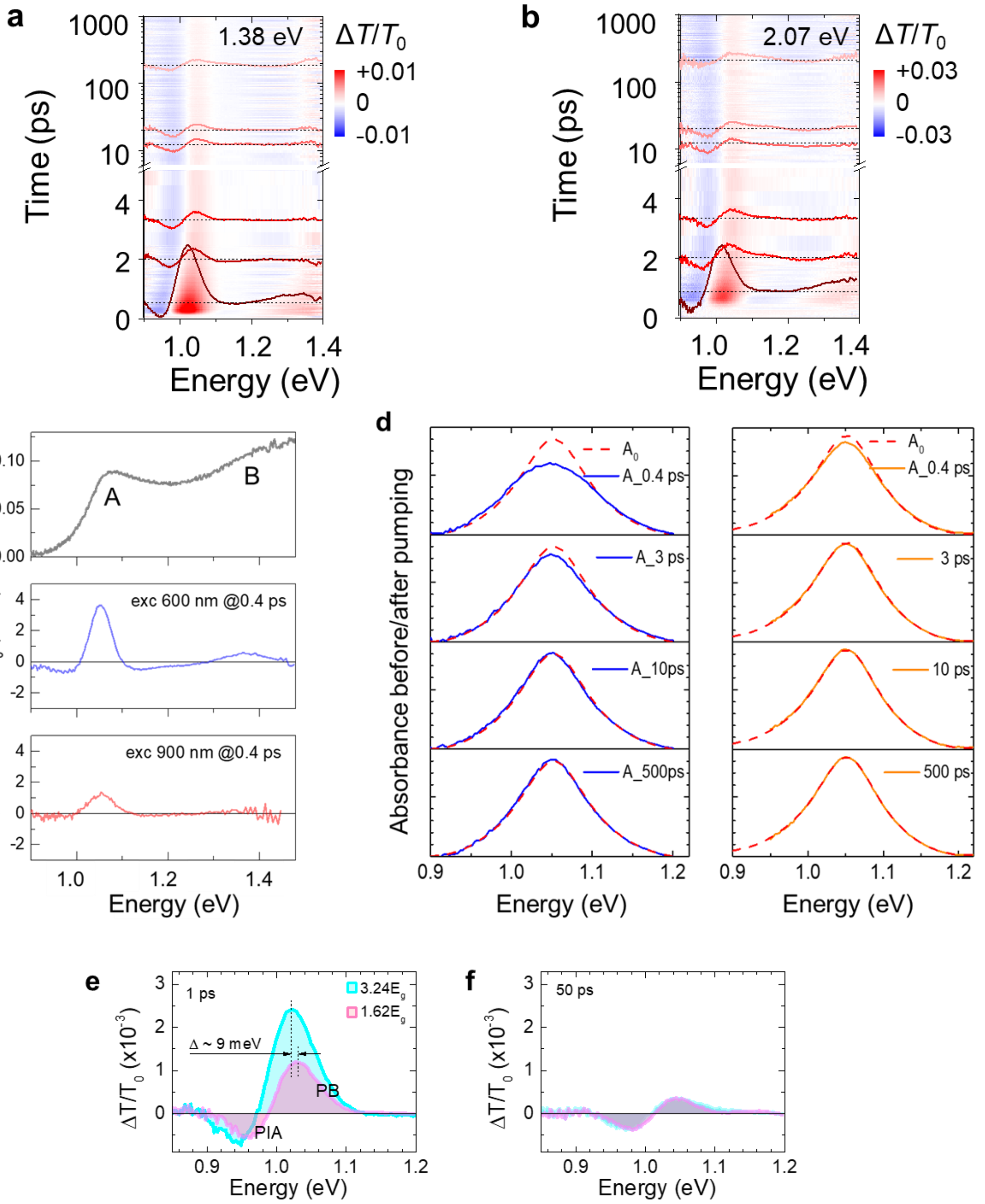

Supplementary Figure 4. a-b, Spectrally and temporally resolved differential transmission map at 1.38 eV and 2.07 eV of excitation pump photon energies. **c-d,** Comparison between absorbances with and without pumping. **e-f.** Differential transmittance spectra at above and below $2E_g$ pump photon energies.

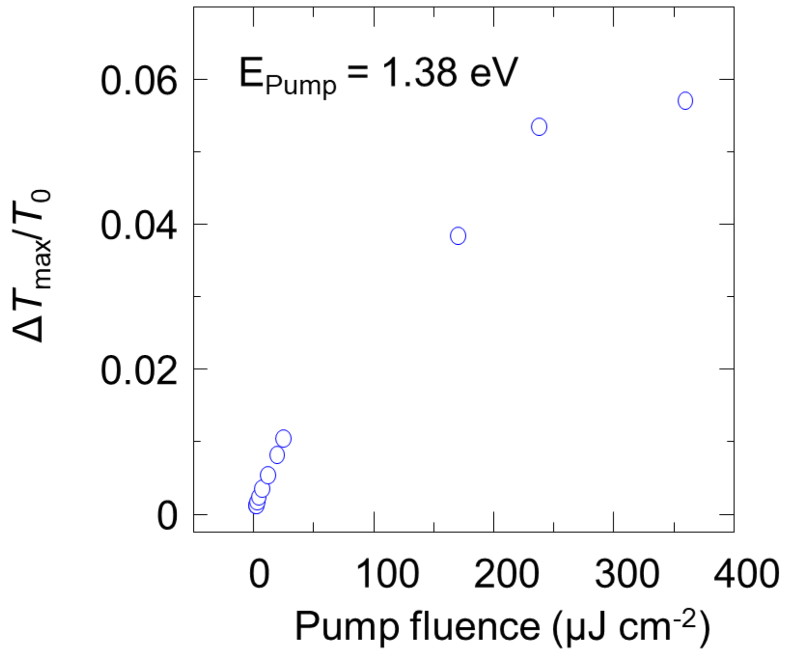

Supplementary Figure 5. The maximum value of ΔT/T0 excited at 1.38 eV is linearly increased up to 1×10^{14} cm^{-2}.

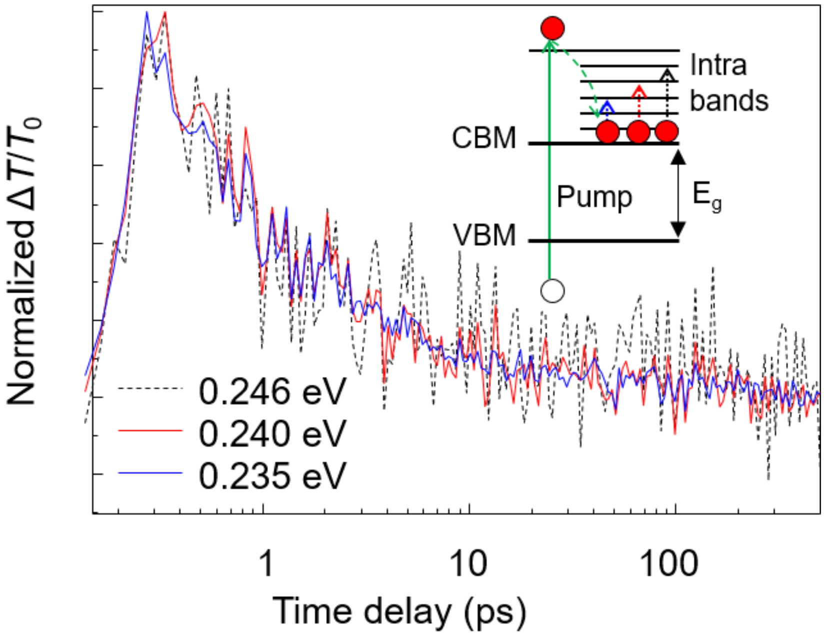

Supplementary Figure 6. $\Delta T/T_0$ with different probe energies normalized by the maximum value. No appreciable difference in the curve was observed between probe energies. We chose 0.24 eV (5200 nm) as a probe energy for induced absorption experiments, because $\Delta T/T_0$ signal is near the maximum as shown in SI Fig. S6 and also its kinetics shows better signal to noise ratio. Ground state electrons at the conduction band minimum obtained from excited electrons by the pump energy are reexcited by small probe energy on to nearly continuum intraband states above conduction band minima (CBM), as shown in the inset. While the maximum $\Delta T/T_0$ varies with probe energy, this plays as a reference only. Quantum efficiency is obtained from the relative change of $\Delta T/T_0$ as a function of flux and pump energy as well. This gives us freedom of choosing different probe energy for induced absorption measurements.

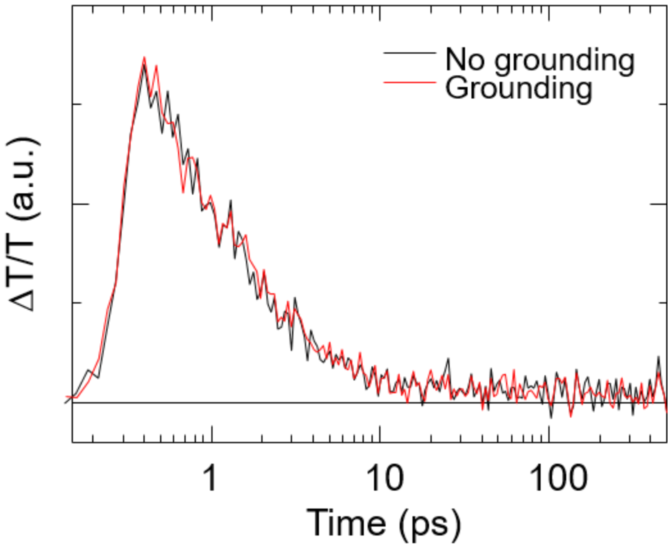

Supplementary Figure 7. Comparison of kinetics for photocharing effect. To eliminate a possibility of experimental artifact owing to charging, TA kinetics under 520 nm excitation with (red line) and without (black line) grounding to the sample were compared; as can be seen, both decay dynamics are identical, thus excluding any effect of charge accumulation in the investigated layer.

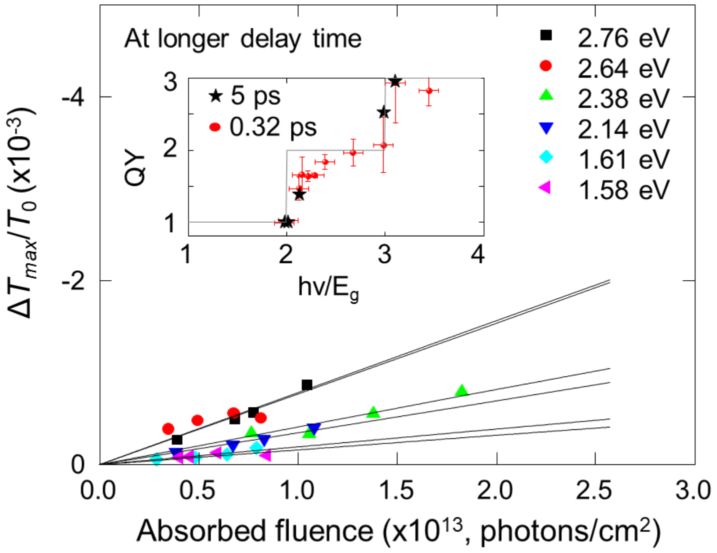

Supplementary Figure 8. $\Delta T/T_0$ as a function of absorbed fluence at different pump photon energies, which were obtained at 5 ps. Compared to Fig. 3b (Main text), the PIA intensity obtained at 5 ps is reduced owing to the possible contribution of electron-phonon interaction. It still shows different slopes at different excitation energies as the CM rate continues to govern over the cooling rate in this time scale.

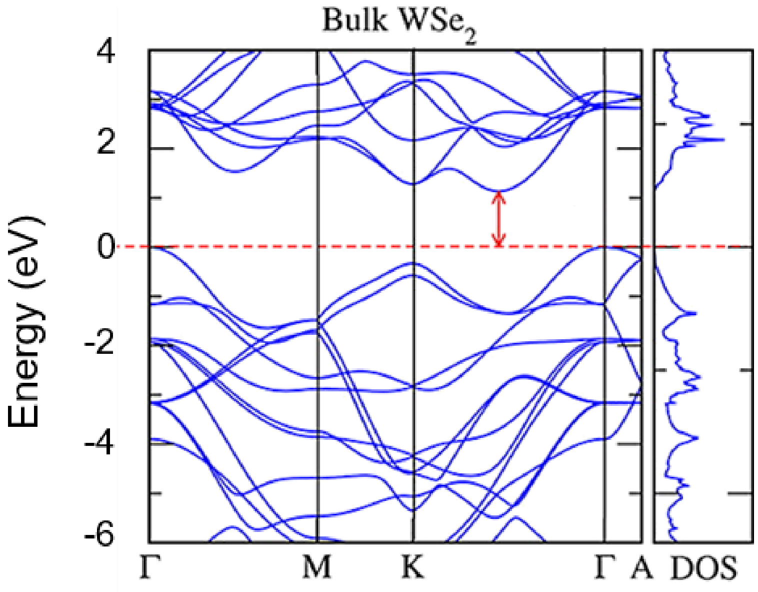

Supplementary Figure 9. Electronic band structure and density of states for bulk WSe_2, adapted with permission from (*Chem. Mater.*, 2015, *27* (4), pp 1278–1284). The red-dashed line indicates the Fermi level. The red arrow is the indirect band gap of WSe_2. Copyright (2015) American Chemical Society[2].

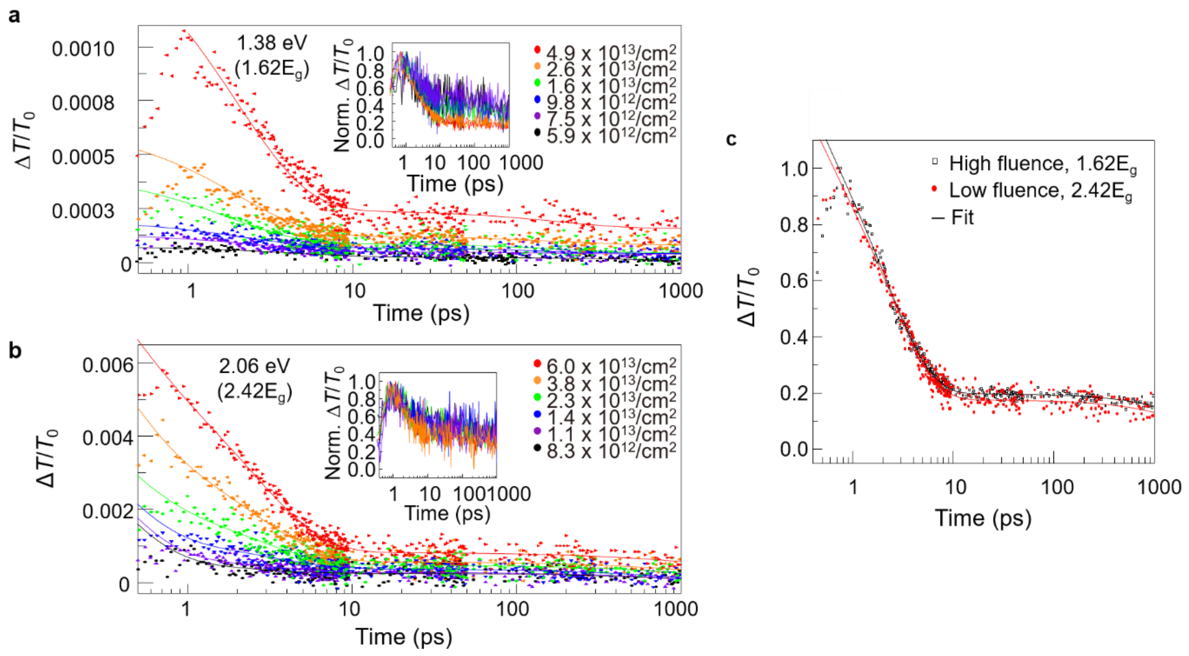

Supplementary Figure 10. Analysis of carrier relaxation PB dynamics. a, Fluence-dependent kinetics excited at 1.38 eV (1.62E$_g$) pump energy in which CM does not occur. The kinetics with four lowest fluencies (black, purple, blue, and green) are characterized by two dominant decay components, τ_f and τ_s. At high fluencies (orange and red), additional faster decay appears with 0.36 ps (τ_A); following recent report (Ref. 25, Main Text) we tentatively identify this component to ultrafast defect-mediated Auger process appearing in vdWaals materials at high carrier densities. The solid lines are 2nd or 3rd order exponential fitting curves. Normalized kinetics are displayed in the inset and the additional faster decay component is clearly shown for high fluence. **b**, Fluence dependent kinetics excited at 2.06 eV (2.42E$_g$) pump energy. All kinetics are characterized by three decay components, τ_1, τ_2, and τ_3. The solid lines are 3rd order exponential fitting curves. The kinetics with normalized intensity is shown in the inset. **c**, Comparison of carrier relaxation dynamics between below 2E$_g$ excitation with high fluence and above 2E$_g$ excitation with low fluence. As can be seen, the normalized two kinetics are identical within the experimental error, while the absorbed photon flux was clearly lower for the high-energy pumping. This is consistent with CM increasing the photo-excited carrier concentration for sufficiently large photon energy.

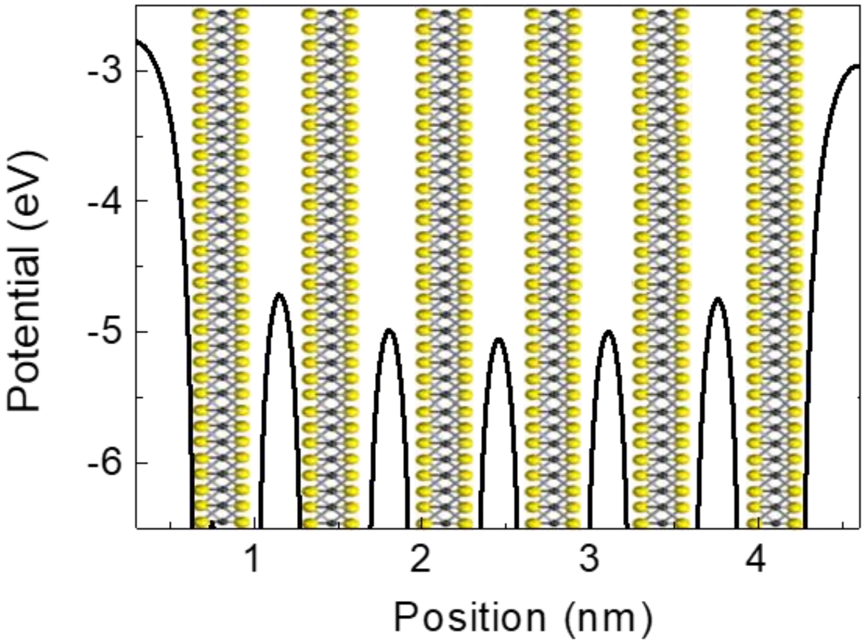

Supplementary Figure 11. Calculated electrostatic potentials of six layers of MoS_2. The vdW layered material is composed of vdW gap as a barrier involving the Coulomb repulsion and metal or chalcogen atom as a quantum well. Therefore, one can expect that the vdW material has the strong confinement effect by the discretized electrostatic potential barrier in extremely narrow region of 3-4 Å between layers. This electrostatic potential barrier of 6L-MoS_2 is calculated by density functional theory (Ref. 31, Main text).

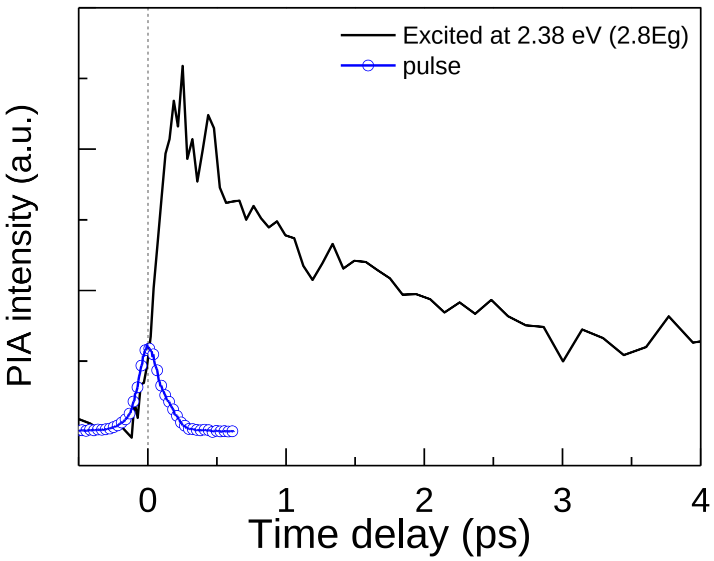

Supplementary Figure 12. Time zero determination. The differential transmittance changes start when pump and probe beams arrive at a focal position simultaneously. In our experimental geometry, the delay stage was inserted in the pathway of the probe beam, and the delay time was acquired owing to the difference of the optical path length between pump and probe. Inserting a KTA (potassium Titanyle Arsenate, KTiOAsO4) crystal at a sample position, we measured the cross-correlation signal for two different wavelengths (visible for pump and infrared for probe) where the experimental geometry is the same as the pump-probe technique. We confirmed that our pulse duration was ~205 ± 20 fs via the cross-correlation measurement

	0D	1D	2D few (mono)	3D
$\tau_{e\text{-}e\ scatt.}$ (ps)	0.05-0.2	0.3	0.02-0.1	0.1 (GaAs), 0.6 (CdS)
$\tau_{e\text{-}ph\ scatt.}$ (ps)	0.5-1	0.7-1.7	0.6 (0.5)	1(GaAs), 6(CdS)
$\tau_{Auger\ recom.}$(ps)	10-300	6-600	60-300 (>800)	
E_b (eV)	0.016-0.31	0.012-0.064	0.7 (1)	0.004-0.027

Supplementary Table 1. Scattering time constants for various materials in 0D[3,4], 1D[5], 2D[5-7], and 3D[5].

Supplementary References

1. Park, J. C. *et al.* Phase-Engineered Synthesis of Centimeter-Scale 1T'- and 2H-Molybdenum Ditelluride Thin Films. *ACS Nano* **9**, 6548 (2015).
2. Kumar, S. & Schwingenschlögl, U. Thermoelectric Response of Bulk and Monolayer MoSe2 and WSe2. *Chem. Mater.* **27**, 1278, doi:10.1021/cm504244b (2015).
3. Schaller, R. D., Agranovich, V. M. & Klimov, V. I. High-efficiency carrier multiplication through direct photogeneration of multi-excitons via virtual single-exciton states. *Nat. Phys.* **1**, 189 (2005).
4. Babentsov, V. & Sizov, F. Defects in quantum dots of IIB–VI semiconductors. *Opto-Electronics Review* **16**, 208 (2008).
5. Wang, F. *et al.* Exciton polarizability in semiconductor nanocrystals. *Nat. Mater.* **5**, 861 (2006).
6. Nie, Z. *et al.* Ultrafast Carreir Thermalization and Cooling Dynamics in Few-Layer MoS_2. *ACS Nano* **8**, 10931 (2014).
7. Wang, H., Zhang, C. & Rana, F. Ultrafast Dynamics of Defect-Assisted Electron–Hole Recombination in Monolayer MoS2. *Nano Lett.* **15**, 339 (2015).
